# Transport and Toxicity of Methylmercury-Cysteine in Cultured BeWo Cells

**DOI:** 10.3390/ijms23010394

**Published:** 2021-12-30

**Authors:** Srividya Ganapathy, Elisa R. Farrell, Simran Vaghela, Lucy Joshee, Earl G. Ford, Olga Uchakina, Robert J. McKallip, Jennifer L. Barkin, Christy C. Bridges

**Affiliations:** 1 Department of Biomedical Sciences, Mercer University School of Medicine, Macon, GA 31207, USA; Srividya.Ganapathy@live.mercer.edu (S.G.); Elisa.R.Farrell@live.mercer.edu (E.R.F.); Simran.Pravinsinh.Vaghela@live.mercer.edu (S.V.); joshee_l@mercer.edu (L.J.); Earl.gilmore.ford@live.mercer.edu (E.G.F.IV); uchakina_o@mercer.edu (O.U.); mckallip_rj@mercer.edu (R.J.M.); 2 Department of Community Medicine, Mercer University School of Medicine, Macon, GA 31207, USA; barkin_jl@mercer.edu

**Keywords:** placenta, syncytiotrophoblast, mercury, oxidative stress, autophagy, toxicology

## Abstract

Mercury is a heavy metal toxicant that is prevalent throughout the environment. Organic forms of mercury, such as methylmercury (MeHg), can cross the placenta and can lead to lasting detrimental effects in the fetus. The toxicological effects of MeHg on the placenta itself have not been clearly defined. Therefore, the purpose of the current study was to assess the transport of MeHg into placental syncytiotrophoblasts and to characterize the mechanisms by which MeHg exerts its toxic effects. Cultured placental syncytiotrophoblasts (BeWo) were used for these studies. The transport of radioactive MeHg was measured to identify potential mechanisms involved in the uptake of this compound. The toxicological effects of MeHg on BeWo cells were determined by assessing visible pathological change, autophagy, mitochondrial viability, and oxidative stress. The findings of this study suggest that MeHg compounds are transported into BeWo cells primarily by sodium-independent amino acid carriers and organic anion transporters. The MeHg altered mitochondrial function and viability, decreased mitophagy and autophagy, and increased oxidative stress. Exposure to higher concentrations of MeHg inhibited the ability of cells to protect against MeHg-induced injury. The findings show that MeHg is directly toxic to syncytiotrophoblasts and may lead to disruptions in the fetal/maternal transfer of nutrients and wastes.

## 1. Introduction

Mercury (Hg) is a naturally occurring heavy metal that is ubiquitous in the environment. While some Hg enters the environment from natural sources, a large fraction of environmental Hg is anthropogenic, i.e., the result of human activity. Coal-fired power plants and small-scale artisanal gold mining are major sources of environmental Hg. Approximately 80% of anthropogenic Hg is released into the air and subsequently deposits in soil and bodies of water. Microorganisms in soil and water methylate mercuric ions to form methylmercury (MeHg). MeHg bioaccumulates and biomagnifies within aquatic species, which leads to high levels of MeHg in the muscle tissue of large, predatory fish. The majority of human exposure to MeHg throughout the world occurs via ingestion of the contaminated fish. The exposure of pregnant women to MeHg is of particular concern given the potential deleterious effect(s) that this toxicant may have on the developing fetus [1]. Despite warnings related to fish consumption during pregnancy, Hg continues to be detected in the blood of pregnant women throughout the world [2,3,4].

MeHg in maternal blood readily crosses the placenta and accumulates in fetal and placental tissues [5,6,7,8,9]. The fetal brain and neurological system are particularly sensitive to MeHg and exhibit injury following exposure to concentrations of MeHg that do not negatively affect the mother [10,11,12]. Interestingly, the concentration of MeHg in cord blood has been shown to be significantly greater than that of the maternal blood, suggesting that fetuses have a higher risk of MeHg intoxication than mothers [13]. Fetal exposure to MeHg leads to many deleterious effects, including cerebral palsy, reduced cognitive function, dysarthria, and strabismus [11,12,13,14]. As MeHg is prevalent throughout the environment and is potentially dangerous to fetal health, it is critical that we have a thorough understanding of the way in which mercuric ions are handled by the placenta.

Although this area is clinically important, little is known about the molecular mechanisms by which MeHg crosses the placenta. Kajiwara et al. [14] reported that cysteine (Cys) conjugates of MeHg (MeHg-Cys) are transported from the maternal blood into the placental syncytiotrophoblasts via a neutral amino acid carrier, which was hypothesized to be system L. Moreover, data from in vivo studies using *Xenopus laevis* oocytes showed that MeHg-Cys is a substrate of both isoforms of system L, LAT1 and LAT2 [15]. Additionally, studies in cultured BeWo cells have also shown that MeHg-Cys is a substrate for LAT1 and LAT2 [16,17]. Given the large number of amino acid transporters present in syncytiotrophoblasts [18], it is likely that a number of the various transport systems are involved in the uptake of MeHg-Cys. Therefore, one goal of the current study was to test the hypothesis that multiple amino acid transport systems are involved in the apical uptake of MeHg-Cys into syncytiotrophoblasts.

Although the toxicological consequences of fetal exposure to MeHg are well documented, there is little information regarding the toxic effects of MeHg on the cells of the placenta. A recent in vitro study in placental trophoblasts demonstrated that exposure to MeHg leads to oxidative stress, cell cycle alterations, and apoptosis [19]. While this study provided some important information about the effects of MeHg on placental cells, we suggest that exposure to MeHg leads to additional, undocumented, toxicological effects in syncytiotrophoblasts. Thus, another goal of the current study was to identify the additional mechanisms by which MeHg induces cellular injury and dysfunction in placental syncytiotrophoblasts.

## 2. Results

### 2.1. Uptake of Cystine and MeHg-Cys in BeWo Cells

**Figure 1:**
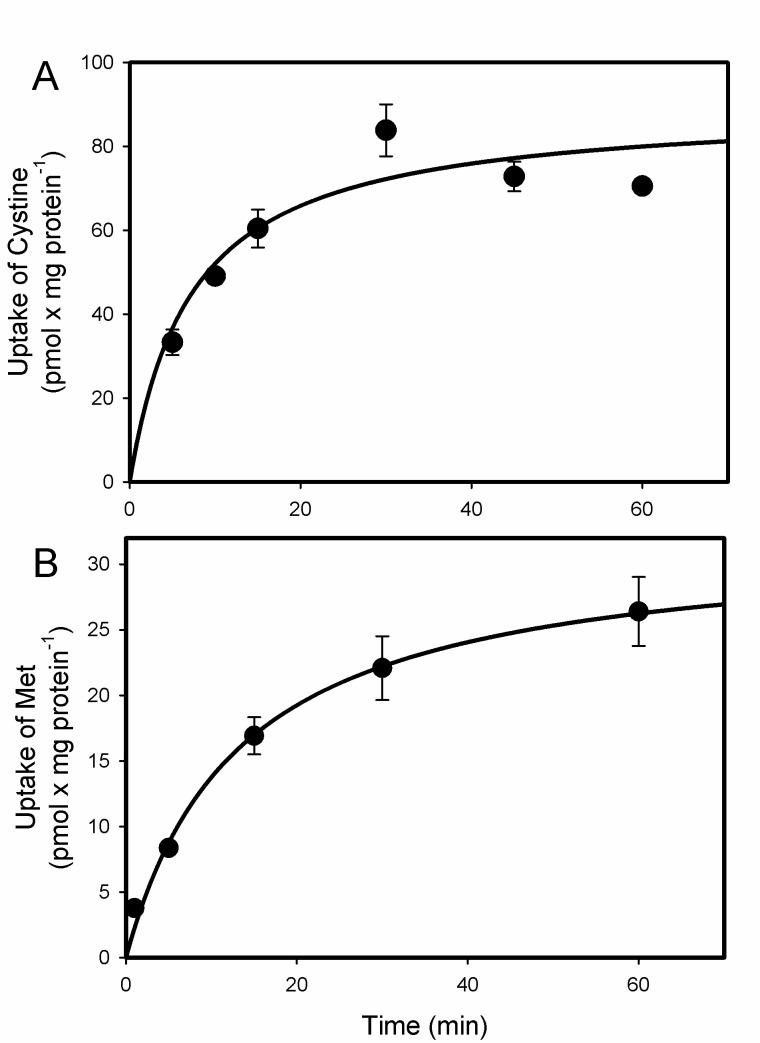
Uptake of 5 µM [^35^S]-cystine (**A**) and 5 µM [^3^H]-methionine (**B**) in BeWo cells. Uptake was carried out at 37 °C for various times between 5 and 60 min. Results are presented as mean ± SE. Data represent 3 experiments performed in triplicate.

The sodium-independent uptake of [^35^S]-cystine was measured over time in BeWo cells (Figure 1A). Cystine (Cys-Cys) is composed of two cysteine (Cys) molecules and is more stable in plasma than Cys. Cys is a critical component of GSH and is necessary for proper fetal development. Moreover, the concentration of cystine in plasma is greater than that of Cys [20]. Therefore, we assessed the uptake of cystine as a measure of normal cellular function. The uptake of cystine was linear during the initial 30 min, after which it began to plateau. The sodium-independent uptake of [^3^H]-methionine was also measured (Figure 1B). The uptake of methionine was less than that of the cystine, but the pattern of the uptake was similar in that it began to plateau after 30 min.

**Figure 2:**
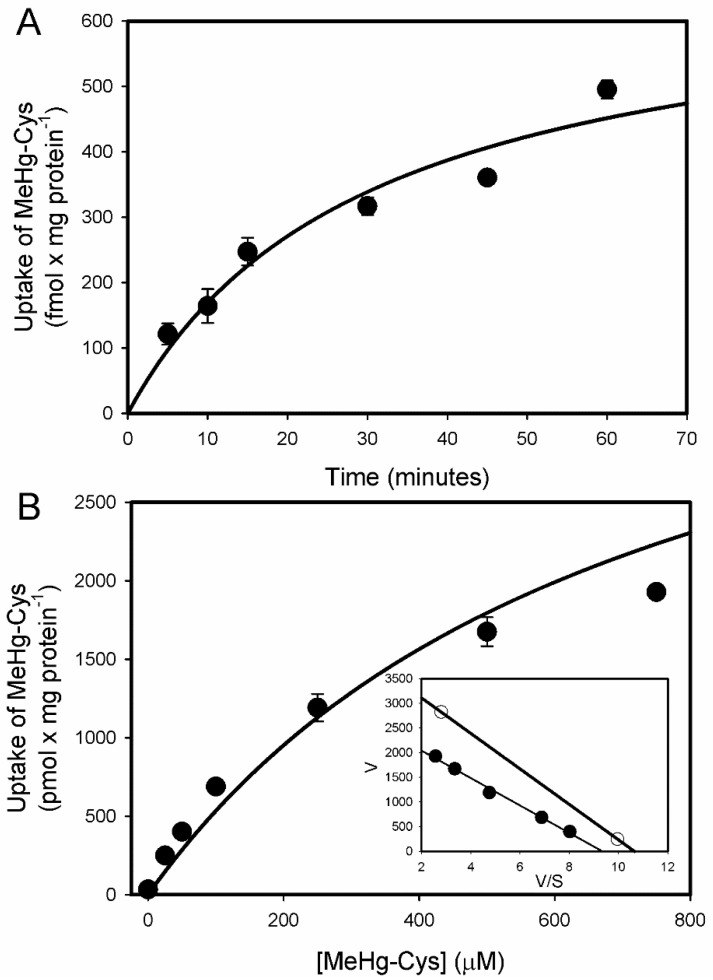
Time course of 5 µM [^14^C]-MeHg-Cys uptake in BeWo cells (**A**). Uptake was carried out at 37 °C for time periods ranging from 5 to 60 min. Saturation kinetics for the transport of [^14^C]-MeHg-Cys (**B**) were assessed in BeWo cells (Inset: Eadie–Hofstee plot). Cells were incubated for 30 min with 5 µM [^14^C]-MeHg-Cys in the presence of unlabeled MeHg-Cys. Results are presented as mean ± SE. Data represent 3 experiments performed in triplicate.

**Figure 3:**
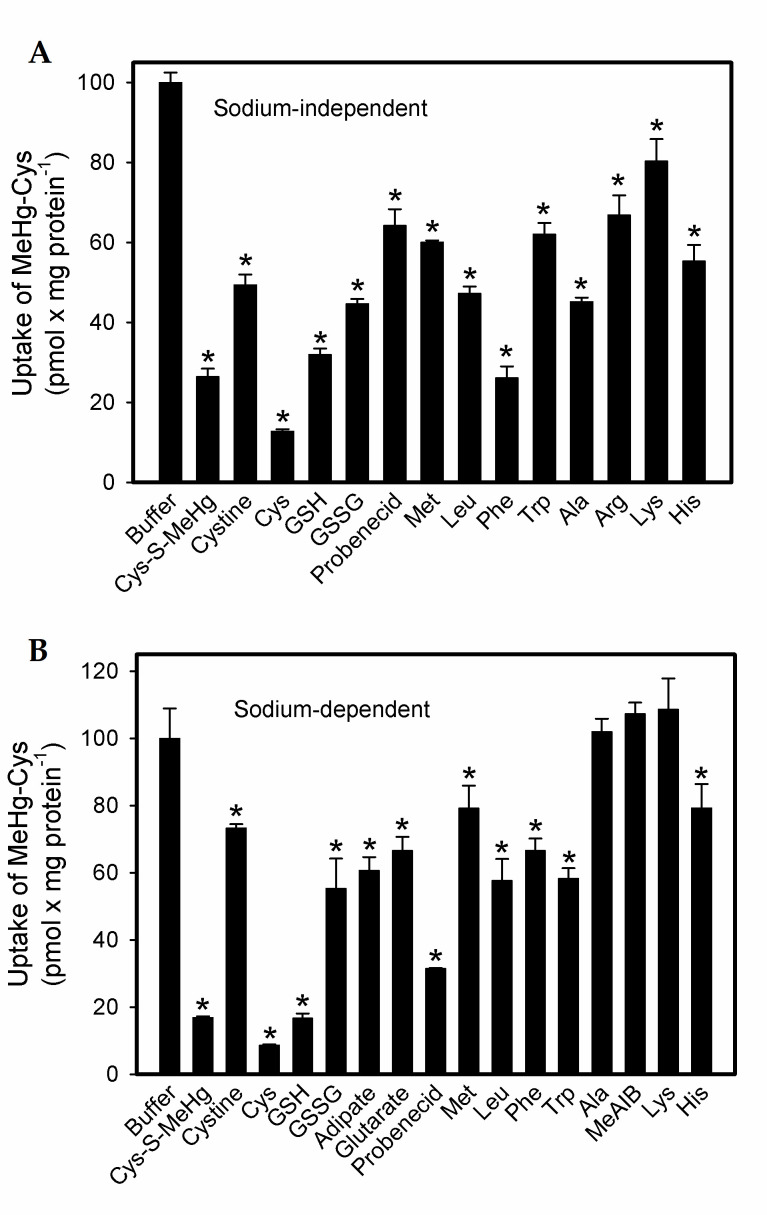
Substrate specificity analyses of [^14^C]-MeHg-Cys transport in BeWo cells. Cells were incubated for 30 min at 37 °C with [^14^C]-MeHg-Cys in the presence of various unlabeled compounds (1 mM Cys-MeHg and cystine; 3 mM all others) under sodium-independent (**A**) and sodium-dependent (**B**) conditions. Results are presented as mean ± SE. Data represent 3 experiments performed in triplicate. *, significantly different (*p* < 0.05) from cells exposed to buffer only.

The sodium-independent uptake of [^14C^]-MeHg-Cys was measured over the course of 60 min in BeWo cells (Figure 2A). As with that of the cystine and methionine, the uptake of MeHg-Cys began to plateau after 30 min. The Michaelis–Menten kinetics of the MeHg-Cys uptake were also assessed in the BeWo cells (Figure 2B). The V_max_ was calculated to be 5.3 ± 2.2 mM and the K_m_ was estimated to be 1.1 ± 0.7 mM. The data were also plotted in an Eadie–Hofstee plot, which suggests that at least two independent transport systems are involved in the sodium-independent uptake of MeHg-Cys (Figure 2B, inset). Substrate specificity analyses of MeHg-Cys uptake were carried out under sodium-independent (Figure 3A) and sodium-dependent (Figure 3B) conditions. The uptake of MeHg-Cys was significantly reduced by the presence of various unlabeled organic anions and amino acids (1 mM MeHg-Cys and cystine; 3 mM all others) under sodium-dependent and sodium-independent conditions.

### 2.2. Cell Viability and Function Assays

**Figure 4:**
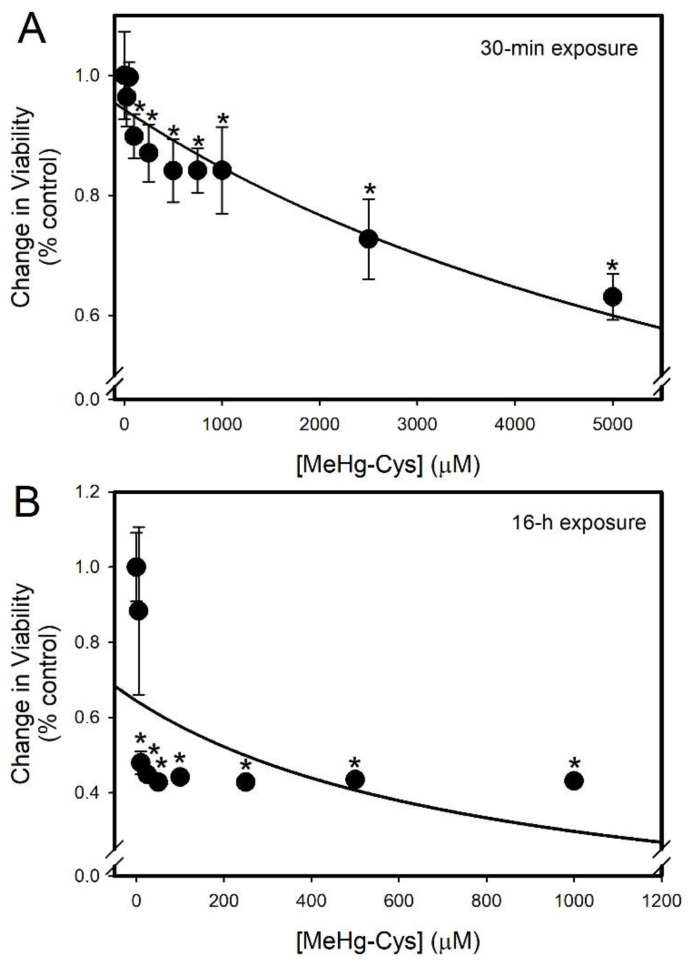
Cellular viability of BeWo cells exposed to MeHg-Cys. BeWo cells were exposed to various concentrations of MeHg-Cys for 30 min (**A**) or 16 h (**B**) at 37 °C and cell viability was measured using a methylthiazoletetrazolium (MTT) assay. The concentration of MeHg-Cys was reduced for the 16 h exposure because higher concentrations resulted in overt cell death. Results are presented as mean ± SE. Data represent 3 experiments performed in triplicate. *, significantly different (*p* < 0.05) from unexposed cells.

An MTT assay was used to measure the viability of the BeWo cells exposed to various concentrations of MeHg-Cys for 30 min (Figure 4A) or 16 h (Figure 4B). The concentrations for the longer exposure period were lower than those used for the shorter exposure because of the cellular toxicity associated with the longer exposure. The viability decreased significantly following the 30 min exposure to 100 µM MeHg-Cys and the higher concentrations. When the cells were exposed to MeHg-Cys for 16 h, a significant decrease in viability was observed following exposure to concentrations of MeHg-Cys as low as 10 µM.

**Figure 5:**
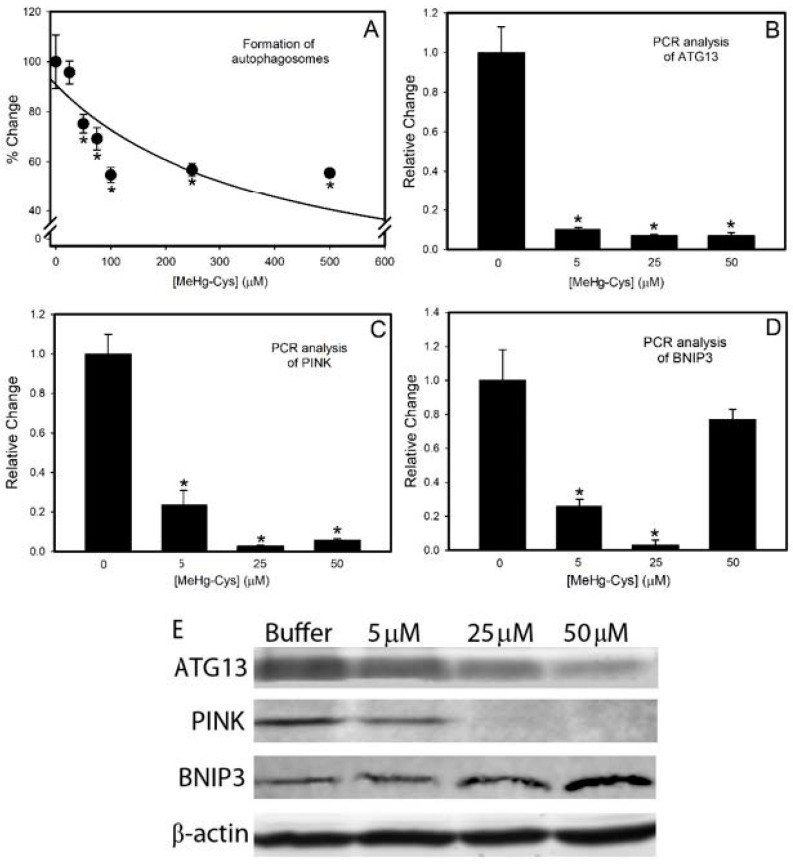
Indicators of autophagy in BeWo cells. BeWo cells were exposed to various concentrations of MeHg-Cys for 30 min at 37 °C, and the formation of autophagosomes was measured (**A**). For analyses of mRNA expression and protein levels, cells were exposed to MeHg-Cys for 16 h at 37 °C. RNA and protein were isolated from the control and exposed cells, and quantitative PCR (qPCR) was used to analyze levels of ATG13 (**B**), PINK (**C**), and BNIP3 (**D**). Western blotting (**E**) measured protein levels of ATG13 (72 kDa), PINK (63 kDa), and BNIP3 (22 kDa). β-actin (42 kDa) was used as the control. Results are presented as mean ± SE. Data represent 3 experiments performed in triplicate. *, significantly different (*p* < 0.05) from unexposed cells.

The formation of autophagic vesicles was measured in BeWo cells exposed to various concentrations of MeHg-Cys (Figure 5A). Autophagy declined significantly in the BeWo cells as the concentration of MeHg-Cys increased. The number of autophagic vesicles formed in control cells was significantly different from that in the cells exposed to 50 µM MeHg-Cys or higher. To confirm the results from the Autophagy Detection kit, we also assessed the expression of the mRNA encoding ATG13, a marker of autophagy [21]. PCR analyses showed that the expression of ATG13 decreased significantly in cells exposed to each concentration of MeHg-Cys (Figure 5B). Markers of mitophagy (PINK1, BNIP3L) [22,23] were also measured using quantitative PCR. The expression of PINK1 (Figure 5C) decreased following exposure to MeHg-Cys. The BNIP3L (Figure 5D) expression decreased significantly when the cells were exposed to 5 µM and 25 µM MeHg-Cys. However, when the cells were exposed to a higher concentration (i.e., 50 µM), the expression of BNIP3L was not significantly different from that in the control cells. Western blotting (Figure 5E) demonstrated protein levels for ATG13, PINK, and BNIP3. The ATG13 and PINK1 protein levels decreased as the cells were exposed to higher concentrations of MeHg-Cys. Interestingly, the protein levels of BNIP3 increased following exposure to MeHg-Cys.

**Figure 6:**
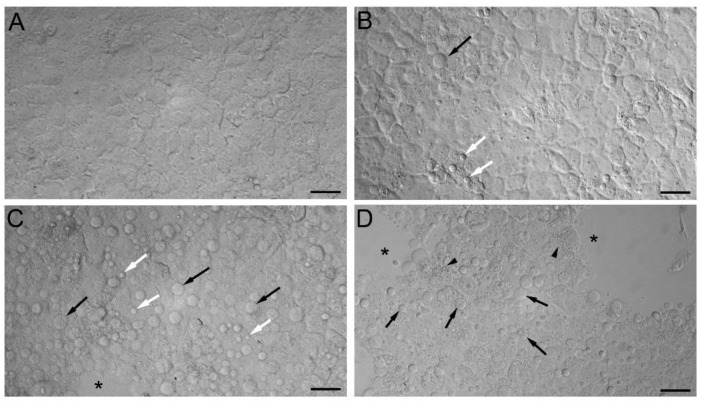
Morphological analyses of BeWo cells were exposed to MeHg-Cys. BeWo cells were exposed to buffer (**A**) or 100 µM (**B**), 250 µM (**C**), or 500 µM (**D**) MeHg-Cys for 30 min at 37 °C. Cells exposed to buffer (**A**) exhibited normal morphology while cells exposed to MeHg exhibited blebs (white arrows) and detaching cells (black arrows). Gaps (*) in the monolayer were evident in cells exposed to 250 and 500 µM MeHg-Cys. Small organelles (arrowheads) were identified in the cytoplasm of cells exposed to 500 µM MeHg-Cys. Images shown are representative of 3 experiments performed in triplicate. Scale bar = 40 µm.

The BeWo cells exposed to various concentrations of MeHg-Cys were examined for pathological changes. The control cells treated with buffer existed as a complete monolayer and showed no signs of injury (Figure 6A). The cells treated with 100 µM MeHg-Cys were present as a monolayer; yet, a few cytoplasmic blebs were identified on the monolayer surface (white arrows; Figure 6B). In addition, a few cells were rounded up and detached from the monolayer (black arrow). When the cells were treated with 250 µM MeHg-Cys, numerous cells appeared to be rounded up and detached (black arrows). In addition, numerous cytoplasmic blebs were present on the surface of the monolayer (white arrows). Gaps (*) in the monolayer were evident throughout the slide (Figure 6C). When the cells were treated with 500 µM MeHg-Cys, numerous cytoplasmic blebs were present (white arrows). Moreover, many cells appeared to be rounded up (black arrows), and the monolayer had numerous areas (*) with missing cells. In addition, multiple small, dark structures (arrowheads) were visible in several cells (Figure 6D). These structures could be phagosomes, peroxisomes, or lysosomes.

**Figure 7:**
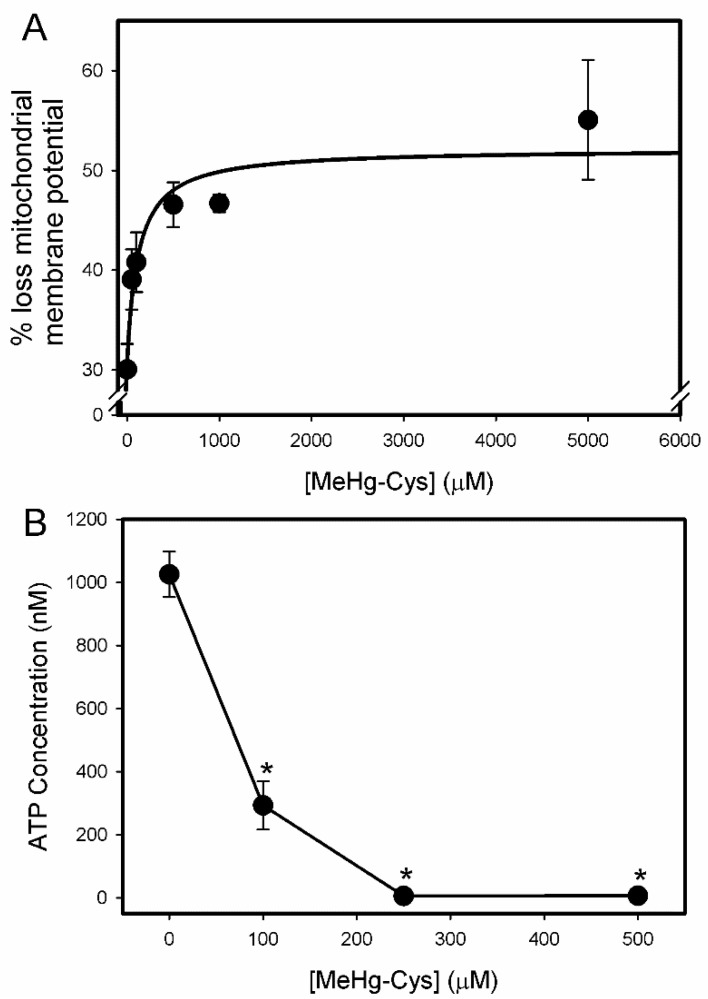
Mitochondrial disruptions in BeWo cells exposed to MeHg-Cys. BeWo cells were exposed to buffer or various concentrations of MeHg-Cys for 30 min at 37 °C. The loss of mitochondrial membrane potential (**A**) was measured using fluorescence-activated cell sorting (FACS). Changes in ATP levels were measured under the same exposure conditions (**B**). Results are presented as mean ± SE. Data represent 3 experiments performed in triplicate. *, significantly different (*p* < 0.05) from unexposed cells.

The changes in mitochondrial membrane potential were assessed by measuring the accumulation of DiOC6 (3) via FACS (Figure 7A). The data are expressed as percent loss of mitochondrial membrane potential. The loss of mitochondrial membrane potential correlated positively with the increasing concentrations of MeHg-Cys. The intracellular concentration of ATP was also measured in the BeWo cells exposed to various concentrations of MeHg-Cys (Figure 7B). As the concentration of MeHg-Cys increased, the concentration of ATP within the cells decreased significantly.

**Figure 8:**
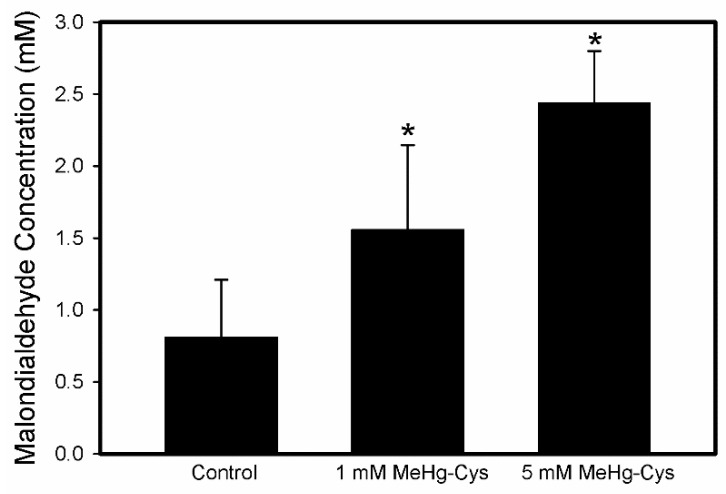
Lipid peroxidation in BeWo cells exposed to MeHg-Cys. BeWo cells were exposed to 1 mM or 5 mM MeHg-Cys for 30 min at 37 °C. Results are presented as mean ± SE. Data represent 3 experiments performed in triplicate. *, significantly different (*p* < 0.05) from unexposed cells.

The amount of malondialdehyde (MDA) in the BeWo cells treated with MeHg-Cys was measured as an indicator of lipid peroxidation (Figure 8). The concentration of MDA increased significantly following treatment of the cells with 1 mM or 5 mM MeHg-Cys.

### 2.3. Markers of Intracellular Injury and Oxidative Stress

**Figure 9:**
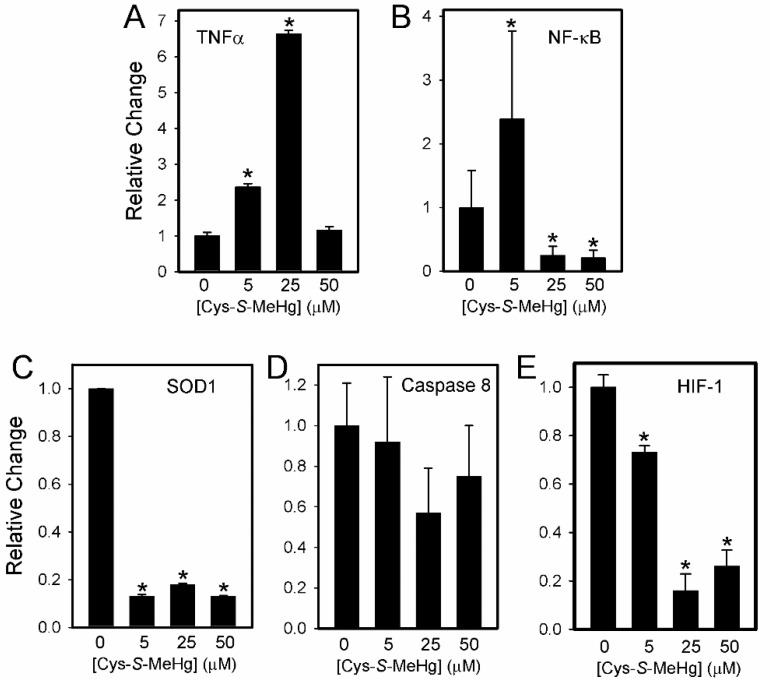
Indicators of oxidative stress in BeWo cells exposed to MeHg-Cys. BeWo cells were exposed to buffer or 5 µM, 25 mM, or 50 µM MeHg-Cys for 16 h at 37 °C. RNA was isolated from the control and exposed cells and quantitative PCR was used to analyze the expression of TNFα (**A**), NF-κB (**B**), SOD1 (**C**), Caspase 8 (**D**), and HIF-1 (**E**). Results are presented as mean ± SE. Data represent 3 experiments performed in triplicate. *, significantly different (*p* < 0.05) from unexposed cells.

The expression of mRNA encoding TNFα, NF-κB, SOD1, caspase 8, and HIF-1 was measured in BeWo cells treated with buffer or various concentrations of MeHg-Cys for 16 h (Figure 9). The expression of mRNA encoding TNFα increased significantly following treatment with 5 and 25 µM MeHg-Cys. Interestingly, when the cells were treated with 50 µM, the expression of TNFα was similar to that in the cells exposed to buffer only. The expression of NF-κB increased significantly in the cells exposed to 5 µM MeHg-Cys but decreased in the cells exposed to higher concentrations. The expression of SOD1 decreased significantly after exposure to each concentration of MeHg-Cys. The expressions of caspase 8 and HIF-1 were similar with the most obvious decrease in expression occurring after the cells were treated with 25 µM MeHg-Cys. Measurement of the GSH and GSSG levels confirmed the presence of oxidative stress (Table 1). The GSH:GSSG ratio decreased as the concentration of MeHg-Cys increased, suggesting changes in the redox balance [24].

## 3. Discussion

Many studies have measured the effects of MeHg on fetal health, but there is a paucity of data related to the direct effects of MeHg on placental syncytiotrophoblasts. Therefore, the current study was designed to examine the means by which MeHg-Cys is transported into cultured placental syncytiotrophoblasts and the toxicological consequences that follow the uptake of this mercuric conjugate.

The current data demonstrated normal uptake of cystine and methionine (Met) in BeWo cells, indicating that these cells are reliable models of functional syncytiotrophoblasts. The uptake of cystine was measured because it is a source of cysteine (Cys), which is an important component of glutathione (GSH), an antioxidant critical for the health of placental and fetal tissues. The uptake of Met was measured because it is similar in size and shape to MeHg-Cys [25]. In addition, Met is a substrate of system L (LAT2), which is thought to play a role in the transport of MeHg-Cys into syncytiotrophoblasts [16,26]. Owing to the numerous amino acid transporters present in syncytiotrophoblasts, we hypothesized that multiple carriers are involved in the uptake of MeHg-Cys from maternal blood. The uptake of MeHg-Cys was inhibited by a variety of amino acids and organic anions under sodium-dependent and sodium-independent conditions, suggesting that multiple carriers are involved in the uptake of this conjugate. Indeed, the current data, when plotted as an Eadie–Hofstee plot, suggest that at least two distinct transport systems are involved in the uptake of MeHg-Cys. Sodium-independent carriers appear to play a greater role in the uptake of MeHg-Cys than sodium-dependent carriers. The ratio of sodium-dependent to sodium-independent uptake was assessed by measuring the extent to which unlabeled MeHg-Cys inhibited the uptake of radiolabeled MeHg-Cys transport in the presence and absence of sodium. The sodium-independent uptake of MeHg-Cys was inhibited by approximately 70% while the sodium-dependent uptake of MeHg-Cys was inhibited by 80%. This finding suggests that only about 10% of MeHg-Cys uptake is mediated by sodium-dependent carriers. In order to identify the specific transporters involved in the uptake of MeHg-Cys, the uptake was measured in the presence of unlabeled amino acids and organic anions. Neutral amino acids (Cys, Met, Leu, Phe, Trp, Ala) inhibited the sodium-independent uptake of MeHg-Cys, suggesting that LAT1 (SLC7A5), LAT2 (SLC7A8), and/or ASC-2 (SLC7A12) may be involved in this uptake. Moreover, uptake was also inhibited by acidic amino acids (Arg, Lys, His), suggesting that y^+^LAT1 (SLC7A7) may also play a role in MeHg-Cys uptake [27,28,29]. The presence of cystine also inhibited the uptake of MeHg-Cys, suggesting that a cystine transporter, such as System x_c_^−^ (xCT; SLC7A11) may also be involved in this uptake [28]. Considering the prevalence of amino acid transporters in placental syncytiotrophoblasts [28], it is not surprising that multiple carriers are capable of mediating the uptake of MeHg-Cys. MeHg-Cys uptake was also inhibited in the presence of probenecid; thus, an organic anion transporter, such as OATP-E (SLC21A13) or OATP41 (SLCO4A1), which are both present in the placenta [30,31], may be also play a role in this process. Surprisingly, the presence of excess GSH and GSSG also inhibited the uptake of MeHg-Cys. This inhibition could be due to the formation of large MeHg-GSH complexes that are not taken up readily by placental syncytiotrophoblasts. Furthermore, as Ala and MeAIB did not inhibit the uptake of MeHg, it can be concluded that System A does not play a role in the uptake of this conjugate [32].

The susceptibility of placental syncytiotrophoblasts to various concentrations of MeHg-Cys has not been characterized completely. The present data indicate that MeHg-Cys is directly toxic to syncytiotrophoblasts, resulting in reduced cellular viability. The mechanisms by which MeHg-Cys reduces cellular viability in any cell type have not been elucidated fully. The current findings show that the exposure of BeWo cells to MeHg-Cys causes a decrease in mitochondrial membrane potential. Previously published studies, using isolated mitochondria exposed to HgCl_2_, showed that exposure to Hg enhances the permeability of the inner mitochondrial membrane to H^+^ and K^+^ [33]. This causes an inward flux of H^+^ and K^+^ across the mitochondrial inner membrane, and the changes in H^+^ and K^+^ levels within the mitochondria lead to a reduction in mitochondrial membrane potential. This, in turn, causes the opening of the mitochondrial permeability transition pore (MPTP) [33]. The MPTP is also stimulated to open by increases in oxidative stress and intracellular Ca^2+^ levels, which have been shown to occur following exposure to Hg [34,35]. Prolonged opening of the MPTP leads to mitochondrial swelling and injury [36]. Consequently, the stores of intracellular ATP are depleted, the electron transport chain is disrupted, and the production of ATP is decreased [33,37], leading to reduced cellular viability. Indeed, the current data show that the intracellular concentration of ATP decreases following exposure to MeHg-Cys. The current findings suggest that exposure to MeHg-Cys leads to pathological alterations in the mitochondria that cause significant defects in cellular energetics.

Exposure to MeHg-Cys increased the production of reactive oxygen species within the cells, as measured by the production of malondialdehyde, an indicator of lipid peroxidation and oxidative stress [38]. Interestingly, the expression of SOD1 decreased after cells were exposed to MeHg-Cys. A similar trend has been observed in other models and may be due to cellular injury and the resulting defects in enzyme/protein synthesis [39,40]. Decreased expression of the genes that are involved in reducing oxidative stress may enhance the susceptibility of cells to injury and accelerate cell death.

Autophagy and mitophagy are important cellular processes that facilitate the sequestration of cytoplasmic debris or dysfunctional mitochondria, respectively, into autophagosomes [41,42]. The current exposure conditions led to a decrease in mRNA encoding ATG13 and ATG13 protein levels. As ATG13 is required for the formation of autophagosomes, this finding suggests that the autophagy is reduced. One possible explanation for this finding could be that exposure to MeHg-Cys inhibits or causes degradation of the autophagic machinery. Some previous studies, using in vitro and in vivo models, reported a reduction in autophagy following exposure to MeHg [43,44], while other studies have shown that exposure to low doses of MeHg enhances autophagy [45]. These reported differences may be due to variations among the cell types studied, differences in the concentrations of MeHg used for the study, and the exposure time for each study. Similarly, exposure to heavy metals has been shown to induce mitophagy [46]. The current Western blot results suggest that levels of BNIP3, a protein involved in mitophagy, increase following exposure to MeHg-Cys. This finding is similar to that of published studies from cultured placental trophoblasts [46]. Surprisingly, the levels of mRNA encoding BNIP3 did not correspond to the protein levels; however, studies of mRNA–protein ratios suggest that mRNA and protein levels do not always correlate because of differences in protein degradation and mRNA stability [47].In contrast to BNIP3, the protein and mRNA levels of PINK1 decreased following exposure to MeHg-Cys. This finding was unexpected considering that the BNIP3 levels increased. Additional studies are necessary to elucidate fully the effects of MeHg-Cys on mitophagy and autophagy in exposed cells.

Decreased autophagy leads to protein aggregation and additional oxidative stress, which may lead to cell injury and death [48]. The present data assessed lipid peroxidation as a measure of oxidative stress. In cells exposed to MeHg-Cys, the lipid peroxidation, as measured by MDA production, increased in a dose-dependent manner. A decrease in the GSH:GSSG ratio also suggests an increase in oxidative stress. In addition, the expression of TNFα and NF-κB increased following exposure to MeHg-Cys, suggesting the presence of an inflammatory process. These data suggest that MeHg-Cys-induced toxicity may be, in part, due to the production of oxidative stress.

The current histological analyses provide visual evidence of the cellular injury caused by exposure to MeHg-Cys. Exposure to lower doses led to the formation of cytoplasmic blebs and the detachment of some cells from the culture surface. At the highest concentration, large areas of cells were rounded up and/or detached. Interestingly, some cells contained small particulate structures, which could be lysosomes. Additional analyses are necessary to identify all the histopathological changes occurring in syncytiotrophoblasts following exposure to MeHg-Cys.

**Figure 10:**
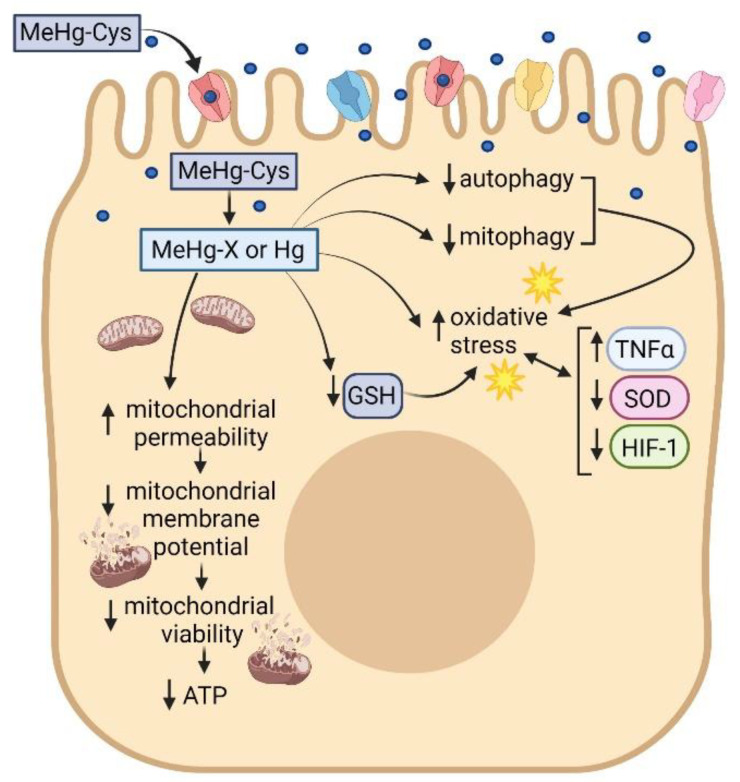
Uptake of MeHg-Cys by placental syncytiotrophoblasts leads to intracellular intoxication characterized by oxidative stress and mitochondrial dysfunction. This figure was created by BioRender.

In summary, the current data show that MeHg-Cys is taken up into the syncytiotrophoblasts using sodium-dependent and sodium-independent mechanisms, such as OATPE, LAT1, LAT2, y^+^LAT1, asc2, and b^0,+^AT. Once inside the cell, MeHg-Cys is directly toxic to syncytiotrophoblasts, causing oxidative stress, inflammation, mitochondrial dysfunction, and eventual cell death (Figure 10). These data represent the first report describing the direct effects of MeHg-Cys on syncytiotrophoblasts.

## 4. Materials and Methods

### 4.1. Tissue Culture

The BeWo cells (ATCC) were cultured in F-12K Medium (ATCC) supplemented with 10% fetal bovine serum (R&D Systems, Minneapolis, MN, USA). The cells were passaged by dissociation in 0.25% trypsin, containing 0.5 mM ethylenediaminetetraacetic acid in phosphate-buffered saline (Life Technologies, Carlsbad, CA, USA). The cultures were maintained at 37 °C in a humidified atmosphere of 5% CO_2_. The cells were not treated with Forskolin, in order to preserve the native transport function within the cells.

### 4.2. Uptake Assays

The uptake measurements were performed as described previously, with minor changes [49,50]. The current experiments measured the uptake of 5 µM [^35^S]-cystine (Perkin Elmer, Waltham, MA, USA), 5 µM [^3^H]-methionine (Perkin Elmer), or 5 µM [^14^C]-MeHg (American Radiolabeled Chemicals, St. Louis, MO, USA), as a conjugate of Cys (MeHg-Cys). The final concentration of the radioactive compound was 1 µCi/mL. MeHg-Cys was created by mixing methylmercury chloride or [^14^C]-MeHg with Cys in a 1:1.25 ratio. The cells were seeded in 24-well plates at a density of 0.2 × 10^6^ cells/well and were cultured for 24 h prior to the experiment. At the time of the experiment, the culture media was removed, and the cells were washed with warm uptake buffer (25 mM 4-(2-hydroxyethyl)-1-piperazineethansulfonic acid (HEPES)/Tris, 140 mM N-methyl-D-glucamine chloride or NaCl, 5.4 mM KCl, 1.8 mM CaCl_2_, 0.8 mM MgSO_4_, and 5 mM glucose, pH 7.5). Uptake was initiated by adding 250 μL of uptake buffer containing the radiolabeled compound of interest. The cells were incubated for 30 min at 37 °C, unless stated otherwise. Uptake was terminated by aspiration of the radiolabeled compounds followed by the addition of ice-cold uptake buffer containing 1 mM sodium 2,3-dimercaptopropanesulfonate (DMPS; Millipore-Sigma, St. Louis, MO, USA) to remove Hg from the outside of the cell [51]. The cells were washed twice with DMPS and were subsequently solubilized with 1% sodium dodecylsuflate (SDS) in 0.2 N NaOH. The radioactivity contained within the cells was measured using liquid scintillation spectrometry.

### 4.3. Cell Viability Analyses

The toxicological effects of MeHg-Cys were quantified using a methylthiazoletetrazolium (MTT) assay, as described previously [50]. This assay measures the activity of mitochondrial dehydrogenase via the conversion of MTT (Millipore-Sigma, St. Louis, MO, USA) to formazan crystals. The BeWo cells were seeded at a density of 0.2 × 10^6^ cells/mL in 96-well plates (200 μL/well). The cells were cultured for 24 h, washed twice with warm uptake buffer and then treated with uptake buffer or MeHg-Cys (25, 50, 75, 100, 250, 500, 750, 1000, 2500, or 5000 μM) in uptake buffer. The incubation was carried out for 30 min or 16 h at 37 °C in a humidified atmosphere of 5% CO_2_. Subsequently, the cells were washed twice with warm uptake buffer and incubated with MTT (0.5 mg/mL) for 2 h at 37 °C in a humidified atmosphere containing 5% CO_2_. Following this incubation, solubilization buffer (10% Triton X-100, 0.1 N HCl in isopropyl alcohol) was added to each well, and the mixture was allowed to incubate for 16 h at room temperature. Each plate was read at 490 nm in a BioTek µQuant spectrophotometric plate reader (Agilent, Santa Clara, CA, USA).

Morphologically discernable pathological changes in BeWo cells exposed to MeHg-Cys were characterized microscopically. The cells were seeded in chambered cover slides (Nalge Nunc, Naperville, IL, USA) at a density of 0.2 × 10^6^ cells/mL (0.5 mL/chamber). The cells were treated with MeHg-Cys (100, 250, 500 µM) in uptake buffer for 30 min at 37 °C. Following exposure, the cells were washed with buffer and microscopic images were captured immediately using an Olympus IX-70 inverted biological microscope (Melville, NY, USA) equipped with Normarsky optics. The images were captured with a Jenoptik Gryphax ARKTUR digital camera.

Fluorescence Activated Cell Sorting (FACS) was also utilized to assess the effect of Hg on mitochondrial membrane potential following exposure to MeHg-Cys. The cells were treated with MeHg-Cys (100, 250, 500, 750, 1000, 5000 µM) for 30 min at 37 °C, following which the cells were trypsinized and exposed to 3,3′-dihexyloxacarbocyanine iodide (DiOC6(3)) and analyzed using FACS.

### 4.4. Autophagy Assay

The formation of autophagosomes in the BeWo cells was assessed using an Autophagy Assay Kit from Millipore-Sigma. The cells were seeded in 24-well plates and exposed to buffer or MeHg-Cys (25, 50, 75, 100, 250, or 500 µM) for 30 min at 37 °C. The cells were washed, and autophagy was assessed according to the manufacturer’s instructions.

### 4.5. ATP Assay

The ATP levels were measured in the BeWo cells exposed to buffer or MeHg-Cys (100, 250, 500 µM) for 30 min at 37 °C. The concentration of MeHg-Cys was increased in order to see detectable changes. The ATP levels were measured using an ATP detection kit from Cayman Chemical according to the manufacturer’s instructions.

### 4.6. TBARS (Thiobarbituric Acid Reactive Substances) Assay

The lipid oxidation was assessed by measuring the concentration of malondialdehyde (MDA) in the BeWo cells exposed to buffer or MeHg-Cys (1, 5 mM) for 30 min. The concentration of MeHg-Cys was increased in order to see detectable changes. The MDA levels were measured using a TBARS assay kit from Cayman Chemical according to the manufacturer’s instructions.

### 4.7. Quantitative PCR

The BeWo cells were exposed to buffer or MeHg-Cys (5, 25, 50 µM) for 16 h at 37 °C. Exposure was extended for these studies to allow for changes in mRNA expression. Following exposure, the cells were washed twice in buffer containing DMPS. Subsequently, RNA was isolated using TRIzol Reagent (Life Technologies, Carlsbad, CA, USA) according to the manufacturer’s protocol. Reverse transcription of one microgram of RNA was carried out using reverse transcriptase and random hexamers (Life Technologies, Carlsbad, CA, USA). Quantitative PCR analyses of superoxide dismutase 1 (SOD1), caspase 8, tumor necrosis factor alpha (TNFα), nuclear factor kappa-light-chain-enhancer of activated B cells (NF-κB), hypoxia-inducible factor 1 (HIF-1), autophagy-related protein 13 (ATG13), PTEN-induced putative kinase 1 (PINK1), and BCL2 and adenovirus E1B 19-kDa-interacting protein 3 (BNIP3L) expression were performed using an ABI Prism 7300 sequence detection system and commercially available gene expression assays (SOD1: Hs00533490_m1; Caspase 8: Hs01018151_m1; TNFα: Hs00174128_m1; NF-κB: Hs00765730_m1; HIF-1: Hs00153153_m1; RIPK3: Hs00179132_m1; ATG13: Hs00207186_m1, PINK1: Hs00260868_m1, BNIP3L: Hs00188949_m1, Life Technologies). GAPDH (Hs02786624_g1; Life Technologies) was used as a reference gene.

### 4.8. GSH/GSSG Assay

Glutathione (GSH) and glutathione disulfide (GSSG) levels were measured using a glutathione assay kit (Cayman Chemical, Ann Arbor, MI, USA). The cells were exposed to uptake buffer or MeHg-Cys (5, 10, 25 µM) for 30 min at 37 °C, and the GSH/GSSG levels were measured according to the instructions included in the kit.

### 4.9. Western Blotting

Western blot analyses were performed on the BeWo cells exposed to culture media, experimental buffer, or MeHg-Cys (5, 10, 25 µM) for 16 h at 37 °C. After treatment, the cells were washed with cold PBS, and subsequently, RIPA buffer (Millipore-Sigma, St. Louis, MO, USA), protease inhibitor cocktail, and phosphatase inhibitor cocktail were added to the cells. The cells were incubated for 5 min at 4 °C, removed from the culture surface by scraping, and lysed in a sonicator for 10 min. The samples were centrifuged for 10 min at 8000× *g* at 4 °C, and the supernatant was collected. The protein concentrations were determined using DC (detergent compatible) protein assay. Sixty µg of protein in loading buffer (LI-COR, Lincoln, NE, USA) with β-mercaptoethanol was loaded into each well of a 10% Tris-HCl gel (Bio-Rad, Hercules, CA, USA). The protein was then transferred to a PDVF membrane (Bio-Rad) using a Criterion blotter. The membrane was incubated in blocking buffer (LI-COR) for 1 h, followed by incubation with rabbit monoclonal antibody against β-actin (1:500; Abcam ab8227, 42 kDa), ATG13 (1:1000; Abcam ab201467, 72 kDA), PINK1 (1:500; Abcam ab216144, 63 kDa), or BNIP3L (1:500; Abcam ab109362, 22 kDa). The membrane was washed and subsequently incubated with goat, anti-rabbit IgG (1:1000; LI-COR). After washing, the membrane was visualized with the LI-COR Odyssey CLx.

### 4.10. Statistical Analyses

The data were first analyzed with the Kolmogorov–Smirnov test for normality and then with Levene’s test for homogeneity of variances. Subsequently, the data were analyzed using a one-way analysis of variance (ANOVA) to assess differences among the means. When statistically significant *F*-values were obtained with ANOVA, differences among means were analyzed using Tukey’s *post hoc* multiple comparison test. A *p*-value of ≤0.05 was considered statistically significant. Each experimental group contained three replicates, and each experiment was performed a minimum of three times (*n* = 9). Four randomly selected microscopic fields (200×) in each culture chamber were examined for the microscopic studies.

## Figures and Tables

**Table 1:** GSH and GSSG concentrations in BeWo cells exposed to buffer or MeHg-Cys.

Treatment	GSH (μM)	GSSG (μM)	GSH:GSSG
Buffer	1.8 ± 0.48	0.21 ± 0.05	9:1
5 μM MeHg-Cys	1.38 ± 0.36	0.31 ± 0.08	5:1 *
25 μM MeHg-Cys	2.49 ± 0.65	0.49 ± 0.13	5:1 *
50 μM MeHg-Cys	0.39 ± 0.10	0.19 ± 0.05	2:1 *

*, significantly different (*p* < 0.05) from cells treated with buffer.

## Data Availability

The data presented in the study are available on request from the corresponding author.
